# Supplementary material for: Functional responses of male and female European green crabs suggest potential sex-specific impacts of invasion
Source: PeerJ. 2023 Jun 2;11:e15424. doi: 10.7717/peerj.15424 (PMC10241166; doi:10.7717/peerj.15424)
Supplement: Supplemental Information 1 [file peerj-11-15424-s001.docx]

**Submission id: a212e391-820d-4eb5-bafc-8801cd17e2aa to IABO**

**Functional responses of male and female European green crabs suggest potential sex-specific impacts of invasion**

Kiara R. Kattler, Elizabeth M. Oishi, Em G. Lim, Hannah V. Watkins, Isabelle M. Côté

**SUPPLEMENTARY INFORMATION**

**Table S1.** Parameter estimates derived from bootstrapped Type II functional response models for male and female European green crabs (*Carcinus maenas*) foraging on varnish clams (*Nuttallia obscurata*). Bca: bias corrected and accelerated bootstrap interval.

| **Sex** | **Parameter** | **Estimate** | **SE** | **Lower Bca bootstrap interval** | **Upper Bca bootstrap interval** | **z-value** | **p-value** |
| --- | --- | --- | --- | --- | --- | --- | --- |
| Male | Attack rate (a) | 2.89 | 0.77 | 1.72 | 5.59 | 3.74 | < 0.01 |
|  | Handling time (h) | 0.12 | 0.02 | 0.07 | 0.17 | 5.79 | < 0.01 |
| Female | Attack rate (a) | 2.01 | 0.54 | 1.09 | 3.64 | 3.73 | < 0.01 |
|  | Handling time (h) | 0.16 | 0.03 | 0.1 | 0.24 | 5.49 | < 0.01 |


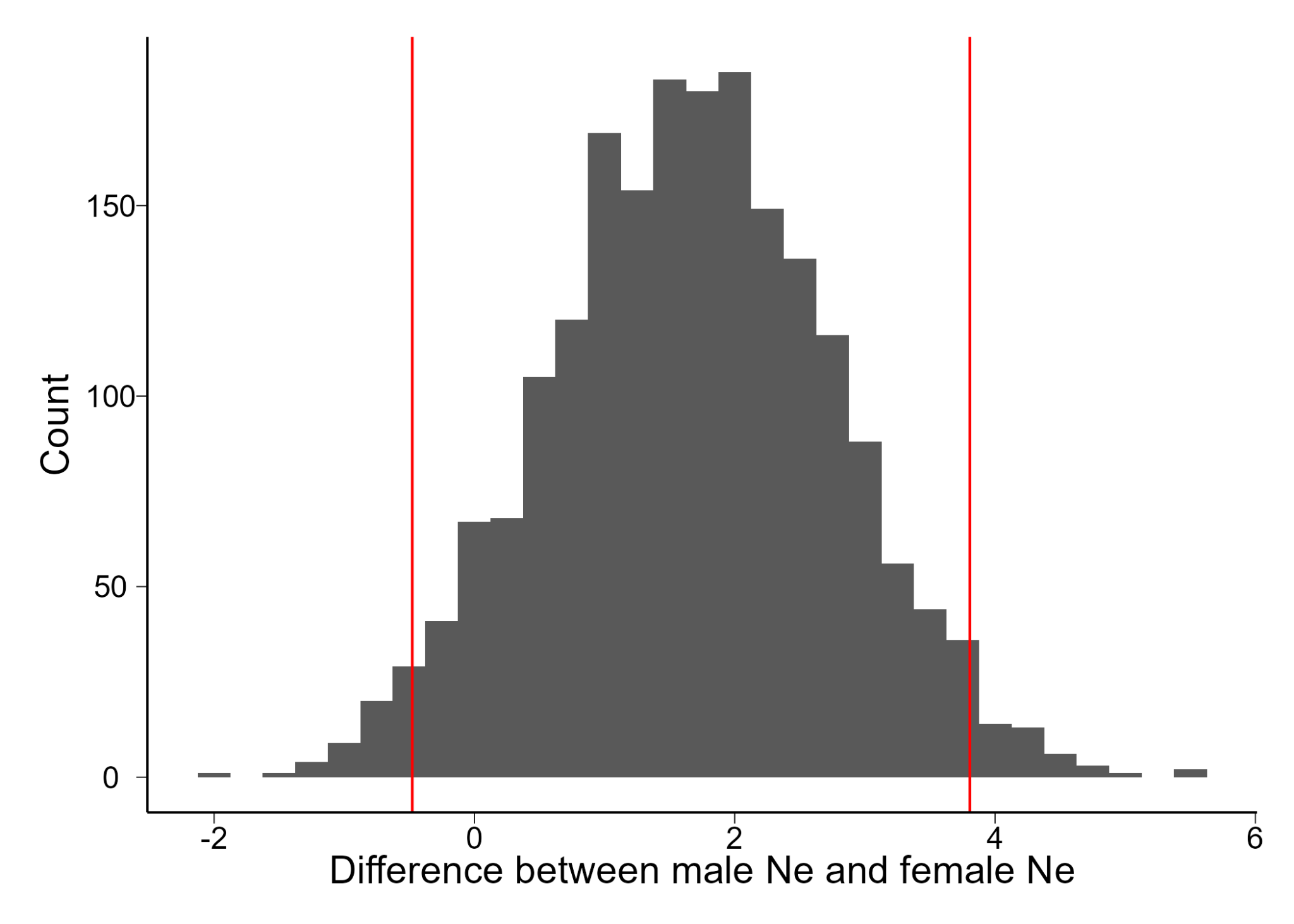


**Figure S1**. Bootstrapped estimates of the difference between the expected number of clams eaten (Ne) by male and female European green crabs (*Carcinus maenas*) at an initial prey density of 16 varnish clams (*Nuttallia obscurata*). The upper and lower limits of the 95% confidence intervals are shown in red.

**
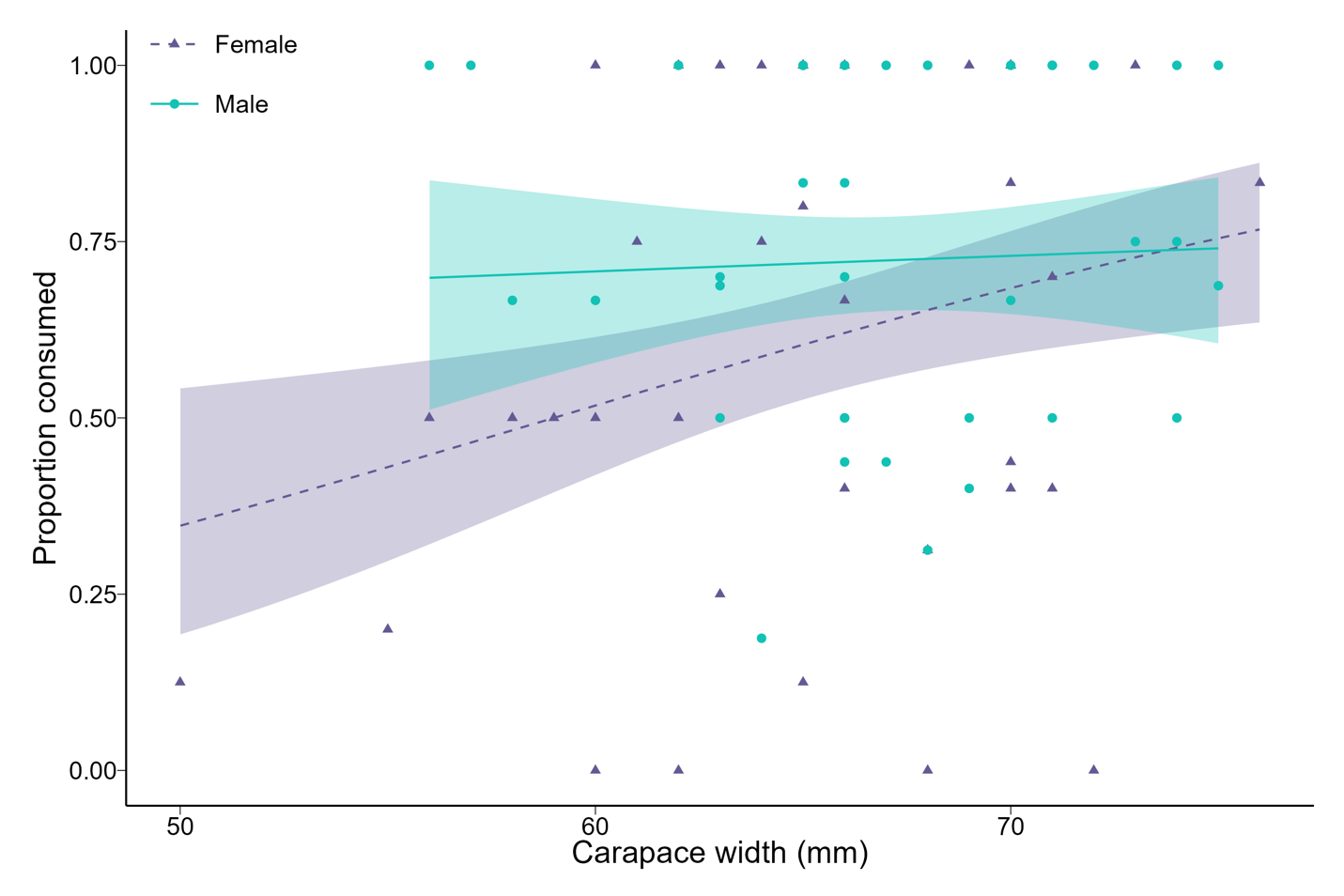
**

**Figure S2.** The proportion of varnish clams (*Nuttallia obscurata*) consumed as a function of carapace width (mm) for female (purple triangles, dashed line) and male (blue dots, solid line) European green crabs (*Carcinus maenas*). The lines are model fit with shaded 95% confidence intervals.


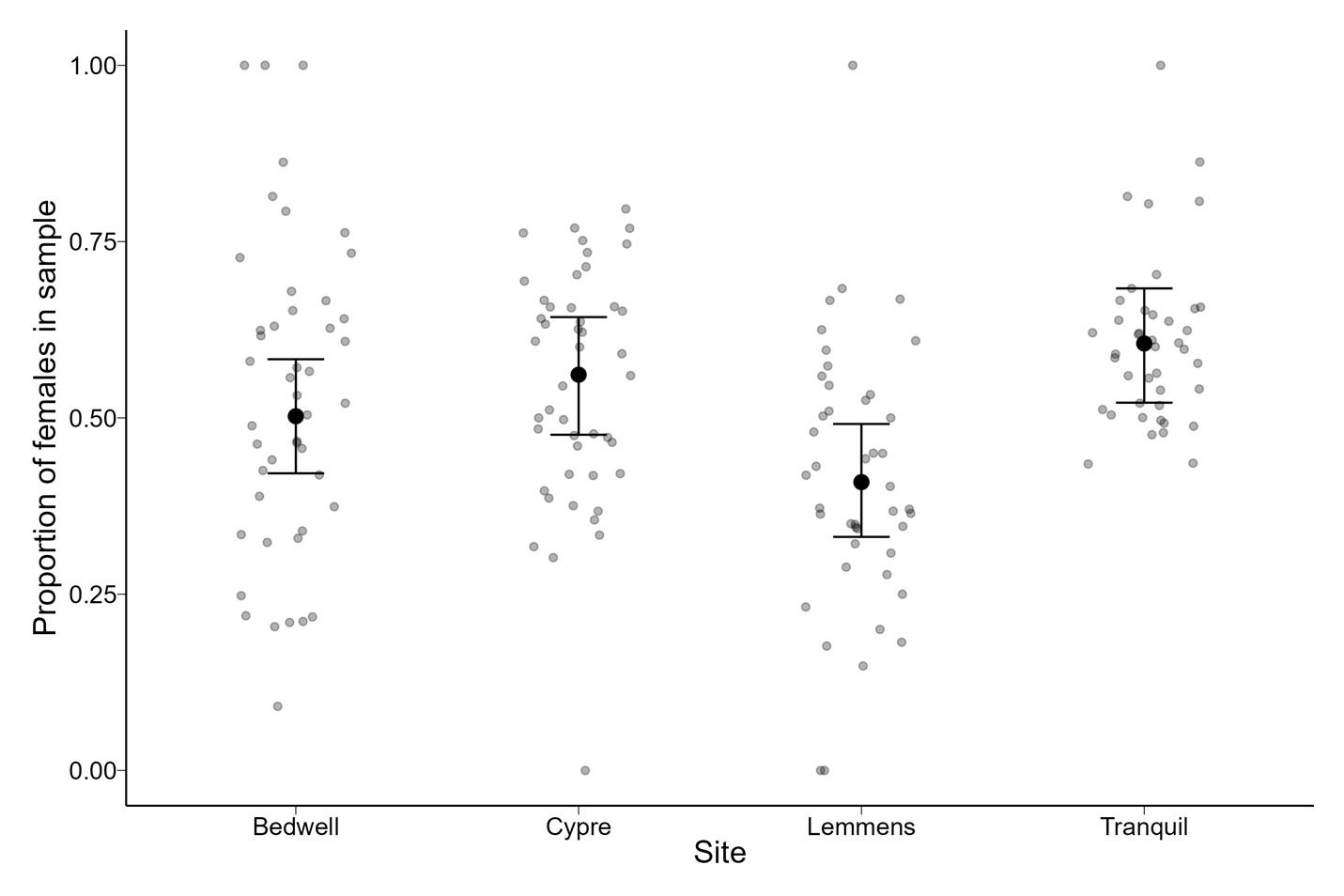


**Figure S3**. Sex ratios (as proportion of females) of four populations of European green crabs (*Carcinus maenas*) sampled by the Coastal Restoration Society as part of the South Coast European Green Crab Control Project. Large points and error bars represent model-fitted means and 95% confidence intervals after accounting for multi-day sampling periods. Grey points represent individual traps.
